# The Relationship Between PM2.5 and Eight Common Lung Diseases: A Two-Sample Mendelian Randomization Analysis

**DOI:** 10.3390/toxics12120851

**Published:** 2024-11-26

**Authors:** Yuhang Jiang, Jingwen Si, Yuhang Wang, Han Zhang, Fang Zhou, Xike Lu, Xin Li, Daqiang Sun, Zheng Wang

**Affiliations:** 1 Department of Clinical Medicine, Medical School, Tianjin University, Tianjin 300072, China; 2 Department of Pathology, Tianjin Central Hospital of Obstetrics and Gynecology, Tianjin 300100, China; 3 Department of Thoracic, Tianjin Chest Hospital Affiliated to Tianjin University, Tianjin 300051, China

**Keywords:** air pollution, PM2.5, COPD, asthma, chronic bronchitis, lung cancer, MR

## Abstract

Air pollutants have both acute and chronic impacts on human health, affecting multiple systems and organs. While PM2.5 exposure is commonly assumed to be strongly associated with all respiratory diseases, this relationship has not been systematically analyzed. This study employed a two-sample Mendelian randomization approach to investigate the effects of PM2.5 on eight common lung diseases, using data from GWAS. Additionally, multivariable Mendelian randomization was applied to assess the direct effects of various air pollutants and the mediating roles of common factors such as BMI and smoking. At a significance threshold of 5×10^−8^, PM2.5 showed a significant causal relationship with both asthma and COPD. When the screening threshold was relaxed to 5× 10^−6^, this exposure continued to demonstrate significant associations not only with asthma and COPD, but also with other respiratory diseases, including pneumonia, emphysema/chronic bronchitis, and lung cancer. In the multivariable Mendelian randomization analysis, which controlled for smoking and bacterial infections, the association with pneumonia became non-significant, while the relationships with the other four diseases persisted. This study provides a systematic exploration of the relationship between PM2.5 and eight pulmonary diseases from a new perspective, deepening our understanding of the impact of air pollution on health and laying the foundation for future efforts to mitigate these effects.

## 1. Introduction

Human activities continuously release various pollutants into the atmosphere, and their accumulation not only threatens the environment, but also has numerous negative impacts on human health. Air pollution is the leading environmental cause of death worldwide, with household air pollution causing from approximately 3.5 to 4 million deaths annually. Women and children living in extreme poverty are the most affected by household air pollution. Ref. [1] These significant changes in atmospheric composition are primarily driven by the widespread combustion of fossil fuels in industries such as power generation and transportation. Given the wide-ranging sources and complex composition of air pollutants, their chemical properties, reactivity, emission methods, and persistence in the environment vary. As a result, the ultimate effects on human health and ecosystems depend on the specific type of pollutant.

Particulate matter (PM) is a broad term for air pollutants composed of a complex and varied mixture of particles suspended in the air. These particles differ in size and composition, originating from both natural processes and human activities [2]. The primary sources of particulate pollution include factories, power plants, and natural dust storms. It can also appear at high concentrations in kitchens. A systematic review by Lin and colleagues [3] provides strong evidence that using gas for cooking and heating can lead to pollutant levels—such as nitrogen dioxide, carbon monoxide, and PM2.5—that exceed the World Health Organization’s indoor air quality guidelines. Particle pollutants vary in size. PM2.5 refers to particles with an aerodynamic diameter of less than 2.5 μm, while PM10 refers to particles with a diameter between 2.5 and 10 μm. Based on these measurements, particles are categorized into ultrafine, fine, and coarse particles. The size of the particles determines where they deposit in the respiratory tract and is closely related to their ability to penetrate the system [4]. PM10 particles primarily deposit in the upper respiratory tract, whereas ultrafine particles can reach the alveoli and even enter the circulatory system.

To date, no single component can fully explain the health effects caused by particulate matter. Factors such as particle size, surface properties, quantity, and chemical composition all play important roles. Particulate matter (PM) is highly complex, as it can adsorb and carry various pollutants. Thus, the composition of PM may differ significantly depending on the environment. There is strong evidence suggesting that ultrafine and fine particles are more hazardous than larger particles (coarse particles) in terms of mortality and cardiovascular and respiratory impacts. Because of their tiny size, they can penetrate deeply into the respiratory system undetected, reaching the alveoli and potentially entering the bloodstream [5]. Consequently, fine particles can cause chronic, long-term effects on the respiratory and immune systems, increasing the risk of disease. Notably, research shows that individuals with asthma, pneumonia, diabetes, respiratory diseases, and cardiovascular conditions are more vulnerable to the harmful effects of particulate matter. Both PM2.5 and PM10 are strongly linked to various respiratory diseases [6]. For example, in the United States, for every 10 μg/m^3^ increase in PM2.5, the incidence rate ratio (IRR) for lung cancer is 1.19 (95% CI: 1.09, 1.30) [7]. In conclusion, PM2.5, due to its small size, poses more significant health risks. These fine particles suspended in the air are also a major contributor to the formation of “smog” in many cities [8,9].

In recent years, rapid industrial development and overpopulation have made China one of the Asian countries facing serious air pollution challenges [10,11].There is evidence suggesting that air pollution may contribute to the onset of asthma, which is also highlighted in ref. [12]. However, not all studies support a definitive causal link between air pollution and asthma. A recent meta-analysis [13] of cross-sectional studies comparing communities with varying levels of pollution found that long-term exposure to pollution did not affect asthma prevalence. Some studies also suggest that environmental air pollution increases the risk of respiratory-related mortality, though the evidence for its impact on lung function and chronic obstructive pulmonary disease (COPD) remains inconclusive [14]. Numerous epidemiological studies, such as ref. [15], have been conducted globally on the health effects of particulate matter. For instance, both short- and long-term exposure to PM2.5 have been positively associated with acute nasopharyngitis [16], a significant correlation has been found between PM2.5 exposure and COPD, and lung cancer mortality has been linked to fine particulate matter exposure [8]. However, most of these epidemiological studies rely on PM2.5 monitors, which lack sufficient spatial resolution. This makes it difficult to accurately measure the actual daily inhalation of PM2.5. Furthermore, the limited placement of monitors within specific study areas or urban regions does not accurately represent the true PM2.5 exposure experienced by the broader population. A recent epidemiological study conducted by the Department of Environmental Health at the Harvard T.H. Chan School of Public Health (Boston, MA) [17] highlighted that the spatial variability in PM2.5 concentrations results in exposure measurement errors. Additionally, in regions with severe PM2.5 pollution, other pollutants are often also present in significant quantities, leading to confounding factors that further diminish the accuracy of study conclusions.

Mendelian randomization (MR) is a statistical method that utilizes genetic variations as instrumental variables to assess the causal relationship between exposure and outcomes [18]. This approach is considered to be a natural simulation of a randomized controlled trial, free from confounding factors and reverse causality biases. Unlike traditional randomized controlled trials—the gold standard for causal inference—MR assigns individuals based on their genotype, thereby avoiding reverse causality and confounding influences such as ethical, socioeconomic, and measurement biases. Our goal is to employ a two-sample MR analysis to investigate the causal relationship between PM2.5 exposure and eight common lung diseases. Multivariable Mendelian randomization (MVMR), a recent extension of MR, uses genetic variations linked to multiple potentially related exposures to estimate the effect of each exposure on a single outcome, resulting in more accurate insights. MVMR also allows for mediation analysis within the MR framework, making it possible to estimate mediation effects [19]. Mediation analysis can identify the factors that mediate the relationship between exposure and an outcome, enabling targeted interventions to reduce the impact of this exposure. This method retains the advantages of using genetic instruments for causal inference, such as eliminating bias from confounding variables while allowing for the estimation of mediation effects. We hypothesize that PM2.5 may either increase or decrease susceptibility to lung diseases. Our study aims to bridge the current knowledge gap regarding the effects of PM2.5 on common lung diseases, guide prevention and treatment strategies for vulnerable populations, and further validate previous findings, offering more comprehensive preventive measures from a new perspective.

## 2. Materials and Methods

### 2.1. Data Source

The study design and data source for this methodological research were based on a genome-wide association study (GWAS) summary obtained from the OpenGWAS data infrastructure. The data cover PM2.5 air pollution and its association with common lung diseases such as chronic obstructive pulmonary disease (COPD), asthma, pneumonia, bronchitis, emphysema/chronic bronchitis, pulmonary fibrosis, bronchiectasis, and lung cancer. A genome-wide association study (GWAS) is a method used to detect gene variations associated with complex human diseases or traits, and its objective is to reveal the impact of genetic variations on the risk of complex diseases, thereby providing new clues and strategies for prevention, diagnosis, and treatment [20]. OpenGWAS is an open genomic-wide association study (GWAS) data infrastructure designed to provide access to a vast collection of publicly available GWAS datasets. The exposure factor under investigation was PM2.5 air pollution, with a sample size of 423,796 individuals, all of European ancestry. A total of 9,851,867 single-nucleotide polymorphisms (SNPs) were analyzed. The summary statistics for the GWAS were sourced from the MRC-IEU. The original lung cancer data were obtained from the UK Biobank, the pulmonary fibrosis data were sourced from Neale Lab, while the remaining datasets were derived from the MRC-IEU. The UK Biobank aggregates genetic data related to PM2.5 exposure from 423,796 European participants. This study was based on the ESCAPE project (European Study of Cohorts for Air Pollution Effects), which utilized a land-use regression (LUR) model to estimate the PM2.5 pollution levels at participants’ residential addresses [21]. The average PM2.5 concentration in the GWAS data was 9.99 (±1.06) µg/m^3^. This dataset is publicly available from MRC IEU OpenGWAS and MR-Base, with the GWAS-ID being ukb-b-10817. The GWAS pipeline output iwa derived from phesant variables from the UK Biobank.

### 2.2. Screening and Extraction Methods

We extracted a GWAS summary dataset for exposure (Table 1). Following previous studies, we selected single-nucleotide polymorphisms (SNPs) within the gene locus that had a significance level (*p* < 5 × 10^−8^) as instrumental variables. If no valid instrumental variables were identified, the *p*-value threshold was relaxed to 5 × 10^−6^. A key principle of MR is that there should be no linkage disequilibrium (LD) among the selected instrumental variables, as a strong LD can lead to biased results. In this study, we applied clumping (*R*^2^ < 0.001, clumping distance = 10,000 kb) to evaluate the LD between SNPs.

### 2.3. Statistical Methods

The Mendelian randomization (MR) model, which uses genetic variants as instrumental variables (IVs), relies on three core assumptions. ① IV Assumption I (Relevance Assumption): The genetic variant (G) is associated with the exposure (X). ② IV Assumption II (Independence Assumption): The genetic variant (G) is independent of any confounding factors (U). ③ IV Assumption III (Exclusivity Assumption): The genetic variant (G) affects the outcome (Y) only through the exposure (X) and not via any other pathways. Assumptions II and III are collectively referred to as being independent of pleiotropy. Due to the inherent biological characteristics of genetic variants, MR models can be prone to estimation failures, often caused by the inadequate representativeness of genetic variants for the exposure or by pleiotropy. This inadequate representativeness of the genetic variants typically reduces the statistical power of the MR model, while pleiotropy can lead to biased effect estimates.

The relevance of Assumption I was evaluated using the F-statistic [22]. An F-statistic greater than 10 indicates that IV Assumption I is satisfied, meaning that there is no weak instrument bias.

### 2.4. Two-Sample MR Method

We initially performed the Mendelian randomization (MR) analysis using the “TwosampleMR” R package [23,24] with inverse variance weighting [25] (IVW) [26] as the primary method to assess the causal relationship between PM2.5 air pollution and common lung diseases. The IVW method regressed the SNP–outcome associations on the SNP–exposure associations, weighting the results by the inverse of the squared standard errors of the SNP–outcome associations, providing an overall weighted estimate of the causal effect. To ensure the robustness of our findings, we also applied several other MR methods for sensitivity analysis, as follows: MR-Egger regression [27], weighted median [28], and maximum likelihood [29]. Additionally, we employed multivariable Mendelian randomization (MVMR) to account for potential confounders. The MVMR analysis was carried out using the mr_mvivw, mr_mvegger, and mr_mvlasso functions from the “mendelianrandomization” R package.

### 2.5. Sensitivity Analysis Methods

To ensure the accuracy of the results, the MR-Egger regression model was employed to test IV Assumption III (the exclusivity assumption). The intercept of the MR-Egger regression provides an estimate of the average pleiotropic effect of all SNPs under the InSIDE assumption. If the intercept differs from zero, this indicates the presence of horizontal pleiotropy [30]. Horizontal pleiotropy was tested using the mr_pleiotropy_test function from the R package “TwoSampleMR.” In cases where the MR-Egger detected pleiotropy, the MR PRESSO method was used to identify and remove potential outliers. Additionally, a leave-one-out analysis was conducted to determine whether any single SNP was driving the causal effect. The Cochran Q statistic was used to test for heterogeneity among the SNPs. If the *p*-value of the Q statistic is greater than 0.05, this suggests that no heterogeneity exists, and the study results do not need to account for heterogeneity effects. However, if the *p*-value was less than 0.05, a random effects model was applied, with the IVW method serving as the primary reference for the results. Pleiotropy testing was performed using the MR-Egger method, and heterogeneity was assessed through both the IVW and MR-Egger methods. The MR-Egger method was used to estimate the impact of pleiotropy, producing more robust pleiotropy-corrected causal estimates under the assumptions of no measurement error and an instrument strength independent of direct effects [31]. The instrument strength was also evaluated using the following formula to calculate the F-statistic:

(1) $$F=\frac{R^{2}(N-1-k)}{(1-R^{2})k}$$

where *R*^2^ represents the variance in the exposure explained by the selected SNPs, *N* is the sample size, and *k* is the number of instrumental variables. If *F* < 10, it suggests a higher likelihood of a weak instrument bias, indicating a weaker association between the instrumental variable and the exposure.

### 2.6. Analysis Software

All two-sample analyses were conducted using the TwoSampleMR package (v0.5.8) in R software (version 4.2.3, R Foundation). For multivariable analyses, the mendelianrandomization package (v1.0.0) was used in R software (version 4.4.1, R Foundation).

## 3. Results and Analysis

### 3.1. Selection of Instrumental Variables

After removing palindromic SNPs, performing clumping, and harmonizing the data, the number of SNPs associated with the outcomes ranged from one to seven. The *R*^2^ and F statistic values for the exposures are summarized in Table S1. To increase the number of SNPs and improve the stability and accuracy of the analysis, we lowered the extraction threshold to 5× 10^−6^, resulting in the extraction of 58 SNPs in total. The *R*^2^ values and F-statistics for the exposure variables are summarized in Table S1. All F-statistics were greater than 10, indicating no evidence of weak instrument bias and confirming the validity of the SNPs. Each SNP under the 5× 10^−6^ threshold was further screened using LDtrait (https://ldlink.nih.gov/?tab=ldtrait) (accessed on September 10, 2024) to ensure that there were no confounding factors.

### 3.2. Sensitivity Analysis

To assess the robustness of the results, a sensitivity analysis was performed, as detailed in Supplementary Table S2. By examining Cochran’s Q statistic and its *p*-value, heterogeneity was identified in the two-sample Mendelian randomization involving chronic obstructive pulmonary disease (COPD) and emphysema/chronic bronchitis. As a result, we applied the random-effects IVW model for analysis. No outliers were detected in either the MR-Egger or IVW tests during the sensitivity analysis.

The MR-Egger intercept revealed a *p*-value of 0.037 in the two-sample Mendelian randomization analysis with COPD, which is less than 0.05, indicating potential horizontal pleiotropy. However, for all other analyses, the intercept *p*-values were above 0.05, suggesting no evidence of horizontal pleiotropy. We used MR-PRESSO to exclude outlier SNPs, and after their removal, the corrected *p*-value continued to show a significant association between PM2.5 and COPD, with the association becoming stronger. While the MR-PRESSO Global test detected outlier SNPs in some analyses, the MR-PRESSO Distortion test indicated no significant difference between the results before and after correcting for outliers. 

### 3.3. Results

The IVW method was employed as the primary analytical approach to evaluate the relationship between PM2.5 and common lung diseases. To minimize potential confounding factors, we carefully selected SNPs based on stringent criteria and conducted two-sample MR analyses using different instrumental variable extraction thresholds (5× 10^−8^). At the 5× 10^−8^ threshold, seven SNPs were identified for the exposure (particulate matter air pollution (PM2.5) || id: ukb-b-10817) in a genome-wide association study. We performed the two-sample MR analysis with these extracted instrumental variables. However, due to the limited number of SNPs, we adjusted the extraction threshold to 5× 10^−6^ to gather more instrumental variables, aiming to provide a more comprehensive analysis by lowering the threshold.

At a significance threshold of 5× 10^−8^, there was a strong indication of a causal relationship between PM2.5 exposure and both asthma and chronic obstructive pulmonary disease (COPD). Specifically, a weighted median analysis of the PM2.5 dataset and the asthma dataset from MRC-IEU demonstrated a significant association (OR = 1.013224, 95% CI: 1.0030398–1.023512, *p* = 0.01080753). Likewise, a significant link was observed between the PM2.5 dataset and a COPD database comprising 463,010 samples (OR = 1.027142, 95% CI: 1.0035843–1.051254, *p* = 0.0236828). The MR Egger analysis further supported this association with a *p*-value of 0.02063981. While no significant associations were found between PM2.5 exposure and emphysema/chronic bronchitis in the IVW and MR-Egger tests, both the weighted median and weighted mode tests suggested a potential association, though with a weaker statistical power. As a result, a definitive causal link could not be conclusively established in this case.

**Figure 1:**
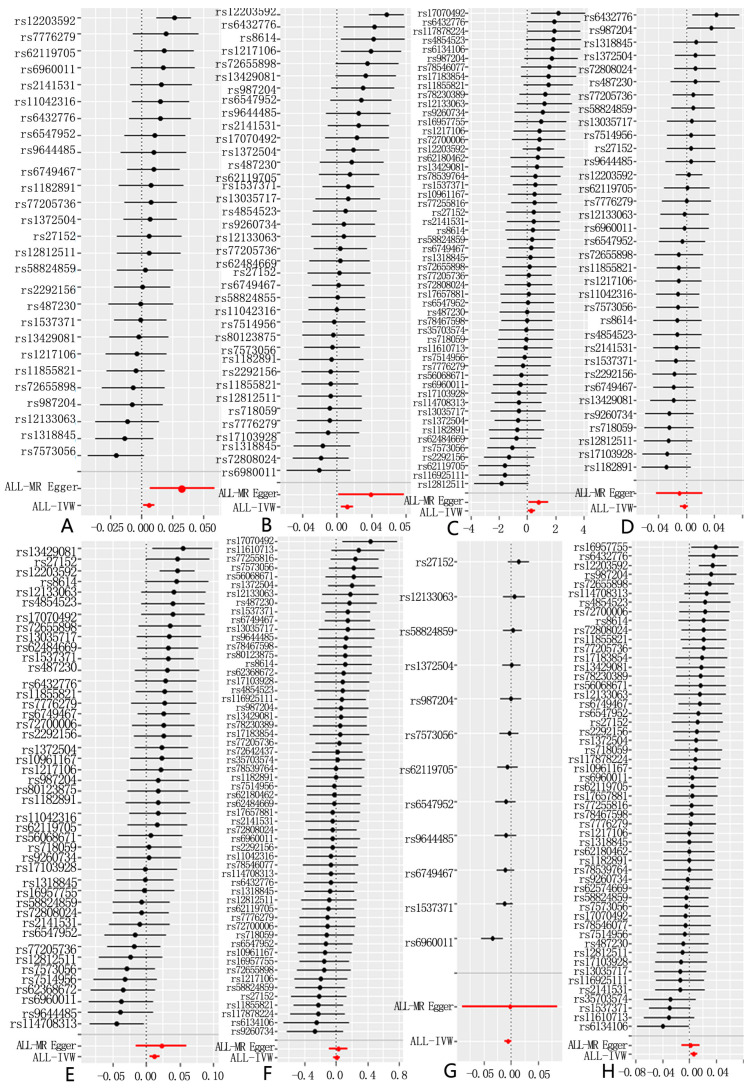
Forest plot of causal associations between PM2.5 and eight common lung diseases in the analysis (5× 10^−6^). (**A**): PM2.5 and asthma, unspecified; (**B**): PM2.5 and chronic obstructive pulmonary disease; (**C**): PM2.5 and pneumonia; (**D**): PM2.5 and bronchitis; (**E**): PM2.5 and emphysema/chronic bronchitis; (**F**): PM2.5 and interstitial pulmonary diseases with fibrosis; (**G**): PM2.5 and bronchiectasis; and (**H**): PM2.5 and lung cancer. NSNP, number of SNPs; OR, odds ratio; IVW, inverse variance weighted; CI, confidence interval.

**Figure 2:**
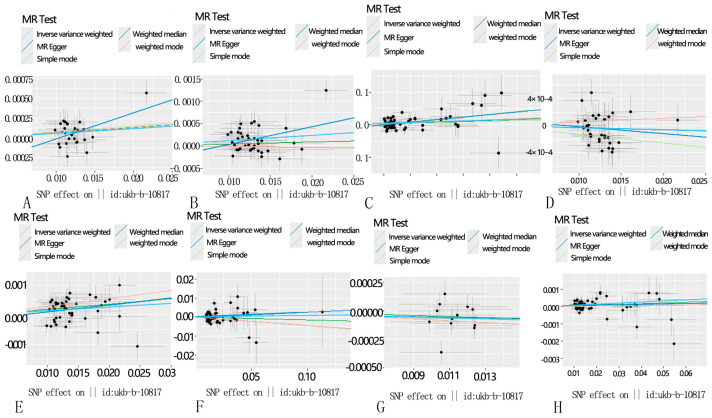
Scatter plot showing the causal estimate for different MRs of PM2.5 on eight common lung diseases. (**A**): PM2.5 and asthma, unspecified; (**B**): PM2.5 and chronic obstructive pulmonary disease; (**C**): PM2.5 and pneumonia; (**D**): PM2.5 and bronchitis; (**E**): PM2.5 and emphysema/chronic bronchitis; (**F**): PM2.5 and interstitial pulmonary diseases with fibrosis; (**G**): PM2.5 and bronchiectasis; and (**H**): PM2.5 and lung cancer.

**Figure 3:**
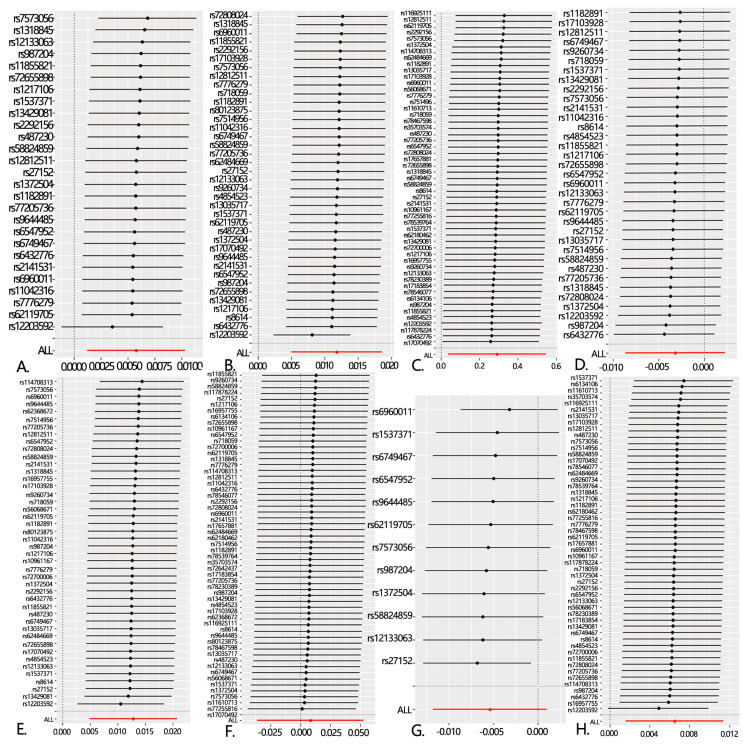
Forest plot of the ”leave-one-out” sensitivity analysis method to show the influence of individual SNPs on the results. The red point indicates the IVW estimates using all SNPs. (**A**): PM2.5 and aAsthma, unspecified; (**B**): PM2.5 and cChronic obstructive pulmonary disease; (**C**): PM2.5 and pPneumonia; (**D**): PM2.5 and bronchitis; (**E**): PM2.5 and emphysema/chronic bronchitis; (**F**): PM2.5 and interstitial pulmonary diseases with fibrosis; (**G**): PM2.5 and bronchiectasis; and (**H**): PM2.5 and lLung cancer.

**Figure 4:**
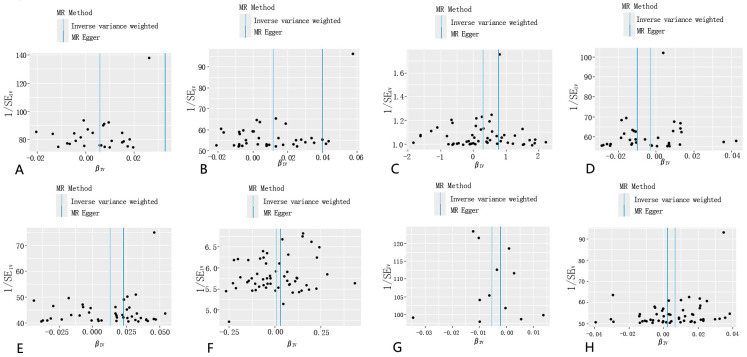
The funnel plot from single SNP analyses for PM2.5 on eight common lung diseases. (**A**): PM2.5 and asthma, unspecified; (**B**): PM2.5 and chronic obstructive pulmonary disease; (**C**): PM2.5 and pneumonia; (**D**): PM2.5 and bronchitis; (**E**): PM2.5 and emphysema/chronic bronchitis; (**F**): PM2.5 and interstitial pulmonary diseases with fibrosis; (**G**): PM2.5 and bronchiectasis; and (**H**): PM2.5 and lung cancer.

However, after performing the MR-PRESSO analysis, the *p*-values for the two-sample Mendelian randomization tests for asthma and chronic obstructive pulmonary disease (COPD) were 0.06338194 and 0.09279563, respectively, indicating no significant association. Interestingly, following the MR-PRESSO analysis, a clear association was found between PM2.5 exposure and pulmonary interstitial fibrosis, without excluding any SNPs, with a *p*-value of 0.03590268. In the two-sample analysis for COPD, Cochran’s Q-test detected heterogeneity, leading to the use of a random-effects IVW-MR model to improve the accuracy of the data analysis. To enhance the accuracy of our analysis, we adjusted the SNP selection criteria by lowering the threshold to 5× 10^−6^. As a result, we successfully identified 58 SNPs associated with respiratory diseases. These SNPs continued to demonstrate significant associations with asthma (OR = 1.005819, 95% CI: 1.0012449–1.010414, *p* = 0.01259716) and chronic obstructive pulmonary disease (COPD, OR = 1.0118626, 95% CI: 1.0049928–1.018779, *p* = 0.0006916272), and also revealed strong links to other respiratory conditions, including pneumonia (OR = 1.337119, 95% CI: 1.0361446–1.72552, *p* = 0.02555535), emphysema/chronic bronchitis (OR = 1.012886, 95% CI: 1.004885–1.02095, *p* = 0.0015536956), and lung cancer (OR = 1.006532, 95% CI: 1.0016285–1.011459, *p* = 0.008972278) (Figure 1 and Figure 2, Table S1). The scatter plot can help to check whether the selected instrumental variable has a sufficiently strong association. If the distribution of points in the scatter plot shows a clear linear trend, it indicates a strong linear relationship between asthma and PM2.5 exposure (Figure 1). Notably, these associations remained statistically significant even after the MR-PRESSO test, further confirming the robustness and reliability of the findings. We conducted a leave-one-out analysis of the IVW results (Figure 3). By removing each SNP individually, we obtained *p*-values of < 0.05, which were consistent with the IVW method’s causal effect estimates, indicating that no single SNP significantly influenced the results. Additionally, the funnel plots for PM2.5 and the eight diseases (Figure 4) were symmetrical, suggesting that pleiotropy did not impact our analysis. The F-statistics, ranging from 20.88 to 69.92 (Table S1), ruled out bias from weak instruments. Given the absence of horizontal pleiotropy, we selected IVW as the primary method for evaluating causal relationships. We conclude that PM2.5 is a risk factor for asthma, COPD, pneumonia, emphysema/chronic bronchitis, and lung cancer. To further explore potential interactions between these SNPs and environmental factors, and to eliminate the influence of other exposures, we performed multivariable Mendelian randomization analyses for smoking and bacterial infections, validated using a LASSO regression model. The results indicated that the association between PM2.5 and pneumonia became non-significant after adjusting for confounding factors, suggesting that PM2.5 may not be a direct risk factor for pneumonia (Table S3). However, the associations with asthma, COPD, emphysema/chronic bronchitis, and lung cancer remained significant. We then investigated potential mediators in the relationship between PM2.5 and these four diseases. Using BMI and smoking as mediators, we found evidence of reverse causality between PM2.5 exposure and these two common factors, indicating that neither BMI nor smoking serves as a mediator in the association between PM2.5 and these common lung diseases.

## 4. Discussion

In the current study, we conducted a Mendelian randomization (MR) analysis to assess the causal relationships between PM2.5 exposure and common lung diseases. Utilizing large-scale summary statistics for PM2.5 and various common pulmonary diseases, our primary MR analysis revealed a significant positive association between PM2.5 exposure and asthma, chronic obstructive pulmonary disease (COPD), pneumonia, emphysema/chronic bronchitis, and lung cancer, consistent with previous studies. We also found no significant association between PM2.5 and bronchitis, interstitial lung disease, or bronchiectasis, an area that currently lacks relevant clinical trials.

Based on the two-sample Mendelian randomization results, PM2.5 exposure showed a clear association with pneumonia, aligning with current mainstream perspectives. DARROW et al. [32] analyzed emergency department visits for bronchiolitis, pneumonia, and upper respiratory infections in children aged 0–4 years from Atlanta and Georgia between 1993 and 2010. They studied the effects of short-term exposure to atmospheric PM2.5 on the emergency department visit rates for pediatric respiratory infections and found that PM2.5 significantly exacerbated upper and lower respiratory tract infections. However, after performing multivariable Mendelian randomization and a LASSO regression analysis adjusting for smoking and bacterial infections, they found no significant association between PM2.5 exposure and pneumonia. This may be related to the complex composition of PM2.5, which contains various bacterial components, and after controlling for relevant confounders, no significant association with pneumonia was observed. It is likely that the induction of pneumonia by PM2.5 mainly relies on its bacterial components, but further research is needed to clarify the exact mechanism. Therefore, this study also emphasizes the validity and necessity of multivariable MR in MR analyses, particularly when a large number of genetic variables are used as instrumental variables. Multivariable Mendelian randomization should be employed to improve the accuracy of experimental results.

Researchers have long been concerned with the effects of PM2.5 on the respiratory system. As early as 2014, TSAI et al. [33] utilized hospitalization data for respiratory diseases from 2006 to 2010 in Kaohsiung, Taiwan, along with atmospheric PM2.5 monitoring data, to explore the relationship between PM2.5 exposure and hospital admissions for respiratory diseases. They found a significant correlation between increased respiratory disease admissions and high PM2.5 concentrations during the colder seasons. The associations between PM2.5 and the eight diseases studied in this article have also been reported in previous research. However, most studies have focused on the relationships between PM2.5 and chronic obstructive pulmonary disease (COPD), asthma, pulmonary inflammation, and lung cancer. The connection between PM2.5 and diseases like pulmonary fibrosis has not received sufficient attention. Observational studies have shown that, in prospective cohorts of both children and adults with asthma, short-term exposure to environmental PM2.5 is associated with asthma symptoms [34]. In a key analysis of COPD prevalence, PM2.5 showed a significant association (OR = 1.52 [95% CI: 1.42, 1.62] per 5 µg/m^3^) [14]. Long-term exposure to PM2.5 has also been significantly linked to an increased risk of lower respiratory tract infections, including a higher pneumonia mortality [35] and chronic bronchitis symptoms. Goobie’s study highlighted that particulate matter smaller than 2.5 μm (PM2.5) is associated with adverse outcomes in idiopathic pulmonary fibrosis, although the exact relationship remains unclear [36]. Lung cancer has been a major focus of research. A meta-analysis of 14 outdoor air pollution studies, mostly conducted in North America and Europe, found that, for every 10 µg/m^3^ increase in PM2.5, the risk of developing or dying from lung cancer rises by 9% (95% CI, 4%–14%) [37].GUO et al. [38] used a spatio-temporal cohort model to assess the relative risk of lung cancer associated with PM2.5 exposure. The results indicated that an increased incidence of lung cancer was significantly correlated with PM2.5 levels. However, there is still a limited understanding of the biological and molecular mechanisms underlying these diseases. There are several hypotheses regarding the impact of PM2.5 on respiratory health, including the oxidative damage theory involving reactive oxygen species (ROS) and the inflammation-related factor activation theory, each supported by their own theoretical framework. Studies have shown that PM2.5 exposure elevates the levels of reactive oxygen species (ROS) [39], which may lead to the development of these diseases by promoting oxidative stress, allergic inflammation, or increasing the efficiency of gene translation in the glycolysis pathway [34,40]. In 2013, Honda et al. [41] collected PM2.5 extracts from industrial and urban areas in Japan. Their study found that organic extracts of PM2.5 could stimulate airway epithelial cells to produce IL-6, thereby triggering an inflammatory response. PM2.5 significantly impacts the occurrence and development of obstructive respiratory diseases, infectious diseases, allergic diseases, and tumors, markedly increasing the incidence and mortality of respiratory diseases. The underlying mechanisms may involve oxidative stress, inflammatory responses, and genotoxicity. However, the effects of PM2.5 on the respiratory system and its mechanisms of action require further investigation.

Our study has several key advantages. To our knowledge, while most existing research on the relationship between air pollution and lung diseases relies on cohort studies, this is the first to systematically use two-sample Mendelian randomization (MR) to explore the causal links between PM2.5 exposure and multiple lung diseases. Previous epidemiological studies have suggested associations between PM2.5 and various common lung diseases, a widely accepted view. However, no prior research has investigated these associations using Mendelian randomization, which helps to address confounding factors and reverse causality. The composition of PM2.5 is often highly complex and frequently coexists with other air pollutants, such as ozone, nitrogen oxides, and sulfur oxides, as shown in ref. [37]. This close interaction with other pollutants can complicate and obscure the understanding of these relationships. PM2.5’s spatial distribution varies, and it often coexists with other pollutants, which can raise doubts about the reliability of past findings.

MR analysis, by using genetic variants as proxies for exposure based on Mendel’s law of independent assortment, strengthens the reliability of results. Since genetic variants exist before the onset of disease, this approach eliminates the influence of reverse causality and provides a long-term perspective on exposure—reflecting an individual’s lifetime biological traits, unlike traditional studies that often focus on short-term exposure. Moreover, by using data from large-scale GWAS meta-analyses, we increased the statistical power of our MR analysis, something clinical trials often cannot achieve. Our findings offer new theoretical and experimental insights into the prevention of air-pollution-related health risks, supporting previous research from a fresh perspective. Additionally, this study provides a foundation for identifying mediating factors, which could further inform strategies for mitigating the impact of air pollution on lung diseases.

Our study has several limitations. First, the GWAS datasets used in the MR analysis were derived from European populations, which may not be representative of other ethnic groups. To enhance the generalizability of our findings, future research should include populations from other regions and countries. Second, we used the LDtrait tool to ensure that the instrumental variables (IVs) met the assumptions of the MR analysis, specifically that the IVs are not directly associated with the lung diseases studied. However, some unpublished SNPs may still be associated with the diseases analyzed, which could affect our results. Our findings were based on a significance threshold of 5 × 10^−6^. While there were not enough SNPs reaching the genome-wide significance threshold of 5 × 10^−8^, we still conducted two-sample MR analyses, and the association between increased PM2.5 levels and asthma and COPD remained consistent with our conclusions. Third, our estimates may be subject to the inherent limitations of MR analysis, such as selection bias. For example, GWAS studies often do not account for the effects of other air pollutants, which may influence the selection of the genetic instruments used in our MR analysis. Since the GWAS data do not provide specific information on the sample’s gender and age composition, the potential impacts of factors such as gender and age have not been further explored in this study. This aspect will be addressed in future work. Fourth, the impact of genetic variants on exposure factors is typically small, meaning that MR analysis may only capture part of the exposure’s effect, making it difficult to fully reflect its influence on disease outcomes. Therefore, further research with larger and more diverse studies is necessary to gather more comprehensive evidence and explore the full range of PM2.5’s effects and mechanisms. It is clear that air pollutants like PM2.5 not only cause short-term, visible harm to the human body, but also have long-term impacts on the respiratory and immune systems. Furthermore, PM2.5 may exert chronic, persistent effects on ecosystems such as water environments, plants, and animals, which, in turn, may cause unforeseen harm to human health. Although these indirect effects may not be immediately apparent, they represent important areas for future research and warrant careful attention. It is believed that, in the near future, with increased participation from various research disciplines and a growing body of studies on PM2.5, our understanding of PM2.5 will be further enhanced. Research on its relationship with respiratory diseases will continue to make new breakthroughs, leading to more practical and effective strategies for preventing and mitigating its harmful effects.

## 5. Conclusions

In summary, our study shows that increased PM2.5 concentrations are significantly associated with a higher risk of asthma, chronic obstructive pulmonary disease (COPD), emphysema/chronic bronchitis, and lung cancer. However, no significant associations were found between elevated PM2.5 levels and pneumonia, bronchitis, interstitial lung disease, or bronchiectasis. Further clinical and foundational research is needed to confirm and strengthen the validity of these findings.

## Figures and Tables

**Table 1:** Outcome and exposure GWAS samples used in this study.

GWAS ID	Trait	Consortium	Sample Size	Year	Category
ukb-b-10817	Particulate matter air pollution (PM2.5); 2010	MRC-IEU	423,796	2018	Continuous
ukb-b-20208	Diagnoses—main ICD10: J45.9 Asthma, unspecified	MRC-IEU	463,010	2018	Binary
ukb-b-16751	Diagnoses—secondary ICD10: J44.9 Chronic obstructive pulmonary disease, unspecified	MRC-IEU	463,010	2018	Binary
ieu-b-4976	Pneumonia	MRC-IEU	486,484	2018	Disease
ukb-b-3467	Non-cancer illness code, self-reported: bronchitis	MRC-IEU	462,933	2018	Binary
ukb-b-7280	Non-cancer illness code, self-reported: emphysema/chronic bronchitis	MRC-IEU	462,933	2018	Binary
ukb-a-358	Underlying (primary) cause of death: ICD10: J84.1 Other interstitial pulmonary diseases with fibrosis	Neale Lab	7637	2017	NA
ukb-b-18163	Non-cancer illness code, self-reported: bronchiectasis	MRC-IEU	462,933	2018	Binary
ieu-b-4954	Lung cancer	UK Biobank	374,687	2021	Disease
ukb-b-19953	Body mass index (BMI)	MRC-IEU	461,460	2018	Continuous
ukb-b-20261	Ever smoked	MRC-IEU	461,066	2018	Binary
ebi-a-GCST90038709	Bacterial infection	NA	484,598	2021	PMID: 33959723

## Data Availability

Publicly available datasets were analyzed in this study. These data can be found in the UK Biobank and Neale Lab.

## Supplementary Materials

The following supporting information can be downloaded at: https://www.mdpi.com/article/10.3390/toxics12120851/s1, Table S1: Causal Estimates of PM2.5 on Common Pulmonary Diseases from Two-Sample Mendelian Randomization. Table S2: Sensitivity analyses of Mendelian randomization for PM2.5 and eight common lung diseases. Table S3: Results of Multivariable Mendelian Randomization.
